# Accurate Determination of Phenotypic Information from Historic Thoroughbred Horses by Single Base Extension

**DOI:** 10.1371/journal.pone.0015172

**Published:** 2010-12-02

**Authors:** Michael G. Campana, C. Mark Whitten, Ceiridwen J. Edwards, Frauke Stock, Angela M. Murphy, Matthew M. Binns, Graeme W. W. Barker, Mim A. Bower

**Affiliations:** 1 Department of Archaeology, University of Cambridge, Cambridge, United Kingdom; 2 Royal Veterinary College, London, United Kingdom; 3 Smurfit Institute for Genetics, Trinity College Dublin, Dublin, Ireland; 4 McDonald Institute for Archaeological Research, University of Cambridge, Cambridge, United Kingdom; Smithsonian Institution National Zoological Park, United States of America

## Abstract

Historic DNA data have the potential to identify phenotypic information otherwise invisible in the historical, archaeological and palaeontological record. In order to determine whether a single nucleotide polymorphism typing protocol based on single based extension (SNaPshot™) could produce reliable phenotypic data from historic samples, we genotyped three coat colour markers for a sample of historic Thoroughbred horses for which both phenotypic and correct genotypic information were known from pedigree information in the General Stud Book. Experimental results were consistent with the pedigrees in all cases. Thus we demonstrate that historic DNA techniques can produce reliable phenotypic information from museum specimens.

## Introduction

One of the promises of historic DNA (hDNA) research, the application of ancient DNA analysis techniques to samples less than approximately 500 years old, has been the ability to reveal phenotypic data normally invisible in the historical, archaeological and palaeontological record. This promise remains largely unfulfilled due to insufficient DNA preservation within historic samples and the absence of efficient techniques for deriving such data. The majority of phylogenetic information and coding genes lie within the nuclear genome. Many of these genes contain single nucleotide polymorphisms (SNPs) that change the expression, structure or function of the proteins they encode, which in turn determine the overall physiology of the organism. Although organisms' phenotypes are not solely determined by their genotypes, genotypic information is a good proxy for phenotypic information in hDNA studies where detailed environmental and ontological information is usually unavailable. It is therefore critical to analyse these SNPs in historic samples in order to improve our understanding of change over time and behaviour in the past. Nevertheless, genotyping of hDNA samples is liable to errors caused by DNA damage and allelic dropout. Therefore, ensuring that SNP-typing methods produce reliable data, with low levels of false alleles and allelic dropout, is crucial in order that inferences can be drawn from hDNA results.

Here, we tested the reliability of a single base extension (SBE) based SNP-typing protocol, SNaPshot™ (Applied Biosystems), on 13 historic Thoroughbred horses for which hDNA results could be verified by comparison with genotypic data inferred from known phenotypes from pedigrees records and contemporary accounts of the horses in question. Although several studies (e.g. [1]–[3]) have utilised SBE to investigate past deceased populations, this is the first to investigate coding SNPs in historic samples for which the correct results are known and verifiable. This information, therefore, permits evaluation of the accuracy of the SBE protocol on historic samples.

Coat colour is one of the most visible, and consequently most studied, genetic systems in horses [4]. We genotyped three SNPs that code for coat colour variation: the agouti signalling protein (*ASIP*), the membrane-associated transporter protein (*MATP*) and the melanocortin-1 receptor (*MC1R*) genes [5]. *MATP* is invariant in Thoroughbred horses and was, therefore, used as an internal control. The obtained genotypes, derived without prior knowledge of the coat colours of the horses, were later compared to the phenotypes recorded in pedigrees. The genotypes were inferred from this pedigree phenotypic data in order to determine the overall accuracy of the SBE technique in historic specimens.

### Coat Colour Markers

In horses, the base coat colour is determined by the relative production of the pigments phaeomelanin (yellow) and eumelanin (brown). This is controlled by the *Extension* (E) and *Agouti* (A) loci, which are encoded by the genes *MC1R* and *ASIP* respectively [6], [7]. The C901T SNP missense mutation in *MC1R* is responsible for the recessive chestnut base coat colour (e allele) [6]. An 11 bp deletion in *ASIP* exon 2 produces the recessive black phenotype (a allele) [7]. Chestnut is epistatic over non-chestnut. The base coat colour is modified by a wide variety of genes that determine various spotting patterns and dilutions. The causative mutations of many patterns remain unknown. The missense G457A mutation in *MATP* causes the incomplete dominant cream dilution (C^cr^ allele) [8]. All Thoroughbred horses are homozygous non-cream (genotype C/C), so this gene was used as a control invariant site. These genes were ideal candidates for testing the SNaPshot™ protocol since coat colour is recorded in studbooks and the genes determine obvious, distinct phenotypes.

## Materials and Methods

### Samples

Sixteen bones and teeth from 13 historic Thoroughbred racehorse specimens were obtained from museum and private collections (Table 1). These specimens were ideal candidates for nuclear DNA analysis since they had previously been shown to have well-preserved mtDNA in concentrations consistent with those expected in historic and ancient samples [9].

**Table 1:** Samples and results of genotyping experiments.

Horse	Birth	Death	Recorded coat colour	Pedigree *ASIP*	DNA *ASIP*	Pedigree *MATP*	DNA *MATP*	Pedigree *MC1R*	DNA *MC1R*	Colour from historic DNA	Pedigree and DNA results match?	Source
Bend-Or	1877	1903	chestnut	A/A	A/A (5)	C/C	C/C (4)	e/e	e/e (4)	chestnut	yes	NHM
Corrie Roy	1878	ca. 1900	bay	A/A or A/a	—	C/C	—	E/e	—	—	no result	NHM
Donovan	1886	1905	black/brown	A/A	A/A (5)	C/C	C/C (4)	E/e	E/e (4)	bay/brown	yes	NHM
Eclipse	1764	1789	chestnut	A/A	A/A (6)	C/C	C/C (6)	e/e	e/e (12)	chestnut	yes	RVC
Hermit	1864	1890	chestnut	A/a	A/a (3)	C/C	C/C (3)	e/e	e/e (4)	chestnut	yes	BLA
Hyperion	1930	1960	chestnut	A/a	A/a (4)	C/C	C/C (3)	e/e	e/e (4)	chestnut	yes	AHT
Ormonde	1883	1904	bay	A/A	A/A* (1)	C/C	C/C (3)	E/e	E/e (3)	bay/brown	yes	NHM
Persimmon	1893	1908	bay	A/a	—	C/C	—	E/e	E/e* (1)	non-chestnut	yes	NHM
Polymelus	1902	1924	bay	A/a	A/a (4)	C/C	C/C (3)	E/e	E/e (4)	bay/brown	yes	ZOO
St. Frusquin	1893	1914	brown	A/a	A/a (3)	C/C	C/C (3)	E/e	E/e (4)	chestnut	yes	NHM
St. Simon	1881	1908	brown	A/a	—	C/C	—	E/E	—	—	no result	NHM
Stockwell	1849	1871	chestnut	A/A	A/A (3)	C/C	C/C (4)	e/e	e/e (4)	chestnut	yes	NHM
William the Third	1898	1917	bay	A/A	A/A* (1)	C/C	C/C (4)	E/e	E/e (4)	bay/brown	yes	NHM

The total number of PCR products SNaPshot™ genotyped is included in parentheses after the genotype. Provisional results, *i.e.* those from a single PCR product, are denoted by an asterisk (*). Genotypes derived from the pedigree records are listed as ‘Pedigree [*Marker*]’. Genotypes obtained by directly analysing the samples' preserved DNA are under the headings ‘DNA [*Marker*]’. No results were obtained for samples marked with a dash (—). Allele nomenclature follows Royo *et al*. [5]. The results from Eclipse's individual skeletal elements were consistent and have, therefore, been grouped together. AHT: Animal Health Trust, Newmarket; BLA: Blankley Stud, Lincolnshire; NHM: Natural History Museum, London; RVC: Royal Veterinary College, London; ZOO: Zoological Museum (University of Cambridge), Cambridge.

### Precautions against Contamination

Strict sterile procedures were followed to ensure the reliability of our results [10]. Pre- and post-PCR procedures were conducted in separate laboratories using dedicated equipment. Personnel were only permitted to move up the DNA concentration gradient. Surfaces were routinely irradiated and cleaned with bleach and ethanol. Non-disposable equipment was decontaminated with bleach, ethanol and UV light. Protective clothing (including face masks, laboratory gowns and double pairs of gloves) was worn at all times. Before extraction, samples were surface-cleaned with 10% bleach, 70% ethanol and 254 nm UV radiation. Filtered pipette tips were used at all times to limit sample aerosolisation and cross-contamination. PCRs were repeated from the same and different extracts. Multiple negative controls, including mock extracts, PCR water blanks, and environmental controls in which a tube was left open throughout a powdering session and subjected to all decontamination procedures to monitor cross-contamination between samples, were included in all experiments [11].

Eclipse, Hermit and Polymelus (Table 2) were independently extracted and the PCR results for *MC1R* were replicated in the Smurfit Institute for Genetics, Trinity College Dublin. These samples were representative of the two genotypes identified in the Thoroughbred data set: Eclipse and Hermit were homozygous chestnut (e/e) and Polymelus was heterozygous non-chestnut (E/e), as his sire, Cyllene, was a chestnut. The one homozygous non-chestnut (E/E) sample, St. Simon, did not yield any amplicons in Cambridge and was, therefore, not replicated in Dublin (see below; Table 1).

**Table 2:** Primers used in this study.

Gene	Forward primer (5′→3′)	Reverse primer (5′→3′)	SNP-typing primer (5′→3′)	Amplicon size (bp)	Reference
*ASIP*	CCTTTTGTCTCTCTTTGAAGC	CAAGGCCTACCTTGGAAG	GATCTCTTCTTCTTTTCTGCT	94	[5]
*MATP*	CTGACCTGGGCCATAAC	CAAATAAGTAGGCTTTGATGGG	CATCAATGAAGTCAGCAGCAAAAT	95 or 84	[5]
*MC1R*	AACCTGCACTCACCCATGTA	AAGATTGCCATCTCCAGCAC	CATCTGCTGCCTGGCCGTGT	92	[6]

### DNA Extraction and Purification

In Cambridge, bone and tooth powder was produced using a Dremel® drilling tool (Dremel Company). For bone samples, surfaces were removed and discarded before harvesting cortical bone. For tooth samples, a root was removed with a Dremel® cut-off wheel (part 540, Dremel Company), and dentine was harvested from the crown so as to minimise damage to the external morphology [12], [13]. Chemical extraction of DNA from the powder followed Kalmár *et al*. [14]. Extracts were purified with a QIAquick PCR Purification Kit (Qiagen) according to the manufacturer's instructions, except that the final elution step was divided into two elutions of 30 and 20 µl.

In Dublin, DNA extractions followed published protocols [15].

### Polymerase Chain Reaction

84 to 95 bp segments of the coat colour genes containing the characteristic SNPs were amplified by PCR (Table 2). PCRs were conducted in 25 µl reactions on Mastercycler® gradient, Mastercycler® ep gradient and Mastercycler® pro (Eppendorf) thermocyclers. Reactions contained 18–19 µl PLATINUM® *Taq* High Fidelity Supermix (Invitrogen), 20 ng BSA (New England BioLabs), 0.8 µM each primer (Table 1) and 2–3 µl DNA extract. For *MC1R*, reaction conditions were as follows: an initial denaturation step of 4 minutes at 94°C, followed by 45 cycles of 1 minute of denaturation at 94°C, 1 minute of annealing at 55°C and 1 minute of extension at 72°C and completed with a final extension period of 10 minutes at 72°C. For *MATP* and *ASIP*, programs consisted of an initial denaturation step at 95°C for 3 minutes, followed by 50 cycles of 20 seconds of denaturation at 95°C, 30 seconds of annealing at 55–57°C and 30 seconds of extension at 72°C, and completed by a 4 minute final extension step at 72°C. PCR products were visualised on 3% agarose gels stained with ethidium bromide.

### SNaPshot™ Genotyping of Coat Colour SNPs

For each sample, at least three PCR products, including amplicons from at least two independent extracts, were genotyped by SBE to ensure accuracy of results. SNaPshot™ genotyping was conducted in London and Cambridge following slightly differing protocols.

#### London Protocol

At the Royal Veterinary College, PCR products were purified using ExoSapIt (Amersham Bioscience) according to the manufacturer's instructions. Purified products underwent SBE using the ABI PRISM® SNaPshot™ Multiplex Kit according to the manufacturer's instructions. After the SBE reaction, the products were purified with SAP according to standard protocols. Purified SBE products were electrophoresed on an ABI PRISM® 3100 automated genotyper.

#### Cambridge Protocol

SBE products were prepared at the McDonald Institute for Archaeological Research. PCR products were first purified using Exonuclease I and SAP according to standard procedures. Purified products underwent SBE using a modified SNaPshot™ reaction. Each 5 µl genotyping reaction contained 1 µl ABI PRISM® SNaPshot™ Multiplex Master Mix, 0.5 µM of the genotyping primer and 1 µl purified DNA. SBE products were electrophoresed on an ABI PRISM® 3730 automated genotyper at the National Institute for Agricultural Botany.

### Confirmation of SNaPshot™ Genotypes

To confirm the SBE genotypes, a subset of PCR products was bacterially cloned with the pGEM®-T EASY kit (Promega) according to manufacturer's instructions. Ten to twelve clones were sequenced per PCR product to determine consensus sequences [16]. For *ASIP*, the difference in size between the two alleles permitted the SNaPshot™ genotypes to be confirmed by comparison with the agarose gel visualisation.

The samples replicated in Dublin were sequenced, and genotypes were derived from sequence traces.

### Pedigree Genotypes

Expected genotypes were derived from coat colour information recorded in the Thoroughbred Pedigree Online Database (www.pedigreequery.com). In addition, photographs and paintings survive for most of the analysed individuals, permitting confirmation of the colours listed in the database. These records are extremely accurate since coat colours were recorded for the analysed individuals, their ancestors and their descendents in the General Stud Book. Non-chestnut individuals, heterozygous for the *MC1R* recessive chestnut mutation, were obligate heterozygotes based on their either having a chestnut parent or producing chestnut offspring. Hetero- or homozygosity for *ASIP* was determined in the same manner. The pedigree results were compared to the genotypes derived experimentally to determine the accuracy of the SNaPshot™ protocol (Table 1).

## Results

In Cambridge, a total of 120 out of 177 PCRs (68%) yielded target coat colour gene products (Table 1).

### *MC1R* Results

*MC1R* SNaPshot™ genotypes, based on at least three PCR products, were obtained for 10 of 13 horses (Table 1). One sample (Persimmon) only yielded *MC1R* products in one reaction and, therefore, his genotyping result must be regarded with caution. Persimmon's provisional genotyping result is marked with an asterisk (*) in Table 1. No PCR products were obtained for samples Corrie Roy or St. Simon. A single control reaction during the *MC1R* experiments produced positive PCR products. This band's sequence matched human *MC1R*, an expected event since the human and horse *MC1R* sequences are highly homologous. Nevertheless, the samples' obtained genotypes from this contaminated experimental set-up were consistent with those from other experiments. Moreover, to verify results, at least four *MC1R* PCR products were genotyped for all samples included in this contaminated experiment except Ormonde.

A total of 111 clones from 12 PCR products representing 10 individuals were sequenced. Cloning results were invariably consistent with SNaPshot™ genotypes.

In Dublin, PCR products from three *MC1R* reactions each for Eclipse and Hermit and six reactions from Polymelus were sequenced. Sequencing results were consistent with SNaPshot™ results obtained in Cambridge.

### *ASIP* Results

*ASIP* genotypes based on PCR products from at least three reactions were obtained for eight individuals (Table 1). PCR products from single reactions were obtained for William the Third and Ormonde. These genotypes must be regarded with caution and are thus denoted by an asterisk (*) in Table 1. No PCR products were obtained for Corrie Roy, Persimmon or St. Simon.

Agarose gel results were consistent with *ASIP* SNaPshot™ genotypes in all reactions except one of Bend Or's five *ASIP* reactions, which was erroneously SNaPshot™ genotyped as heterozygous. This reaction was probably contaminated during post-PCR genotyping preparation, since its agarose gel banding pattern was consistent with Bend-Or's expected *ASIP* genotype (homozygous A/A).

### *MATP* Results

*MATP* genotypes based on PCR products from at least three reactions were obtained for 10 individuals (Table 1). One *MATP* experiment was discarded due to contamination detected in PCR controls. No PCR products were obtained for Corrie Roy, Persimmon or St. Simon. All PCR products were homozygous non-cream (C/C) as expected from phenotypic data, except for one from Eclipse's tooth, which was genotyped as homozygous cream (C^cr^/C^cr^). This one read is probably the result of a C→T transition artefact [17].

### Single Allelic Dropout

Single allelic dropout was observed in 21% of *ASIP* and 26% of *MC1R* genotyping reactions. *MATP*'s single allelic dropout rate was incalculable since all samples were homozygous for this marker.

### Comparison with Expected Genotypes from Pedigree Records

Although individual reactions' genotypes were inconsistent with the expected genotypes derived from pedigree records, all the horses' final experimentally derived genotypes (including provisional results) were consistent with the expected genotypes (Table 1).

## Discussion

We recovered nuclear DNA giving reproducible genotypes from 77% of the historic Thoroughbred samples using SNaPshot™. SNaPshot™ is ideal for analyses of degraded material since it targets the very short (<100 bp) DNA molecules likely to survive in historic samples. Moreover, the final experimentally-derived genotypes were accurate in all cases. SNaPshot™ also detected sequence variants more sensitively than cloning and sequencing (see below). The SNaPshot™ protocol thus proves to be an extremely robust method for deriving nuclear data from historic samples.

### Allelic dropout

Historic DNA studies have been limited in their ability to quantify and address the problem of single allelic dropout, in which one allele from a heterozygous individual does not amplify rendering a false homozygous result. Most studies (e.g. [18]) rely on the repetition of results to verify apparent homozygotes, since the true genotypes of the samples are unknown. The number of repetitions required to verify homozygosity is derived from non-invasive sampling studies [19], whose results may not be applicable to hDNA studies. Since our samples' true genotypes were accurately known, we were able to calculate the exact rates of single-allelic dropout. The observed rates (21% of *ASIP* and 26% of *MC1R* reactions) are relatively low for degraded samples [19]–[23]. Given the observed dropout rates in our samples for *MC1R* and *ASIP*, the probability of detecting heterozygotes by three independent genotyping experiments was greater than 98%. Although these low dropout rates attest to the reliability of the SBE protocol, our historic Thoroughbred samples are extremely well-preserved and more-degraded materials will have higher dropout rates and, consequently, require more replication experiments.

### Characteristics of SNaPshot™ on degraded samples

**Figure 1:**
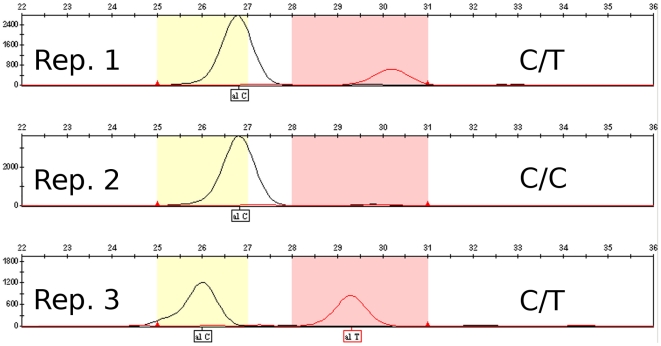
Allele ratios varying between three replicates of the same sample (Ormonde) due to stochastic effects. There is evidence of allelic dropout in the second replicate. The differences in location of the peaks between the repetitions are due to variation between genotyping runs.

When performing the SNaPshot™ protocol on modern heterozygous samples, relative allelic peak heights are typically consistent between repeated amplifications of the same individual. This did not hold true in historic samples. Stochastic effects during amplification varied the observed allele ratios from near equal frequencies to complete dropout of one allele (Figure 1). This pattern is expected in historic samples (e.g. [19]). It also reinforces the need to repeat genotyping experiments, especially on apparent homozygous individuals, since allelic dropout is frequent even in very recent, well-preserved specimens.

Furthermore, SNaPshot™ proved to be robust to the most common form of hDNA damage, C→T transitions [17]. Although these lesions are ubiquitous in hDNA, the probability of a lesion occurring at any one base is very low. Consequently, we observed only one artefact T allele in the 37 *MATP* genotyping reactions on our historic sample set. Nevertheless, the error level due to C→T transitions (3%) is a serious concern in hDNA analyses since, unlike in our study, discovery and correction of these artefacts may prove difficult. This is especially true in poorly preserved specimens where these lesions may occur at higher frequencies than that observed in our samples and where repeated amplification of target SNPs may be difficult. Multiple repetitions of SNaPshot™ results are therefore critical to ensure that these low-frequency artefacts are discovered and corrected. In apparent heterozygotes, both alleles must be replicated multiple times to weed out errors due to C→T transitions.

### SNaPshot™ versus cloning and sequencing

**Figure 2:**
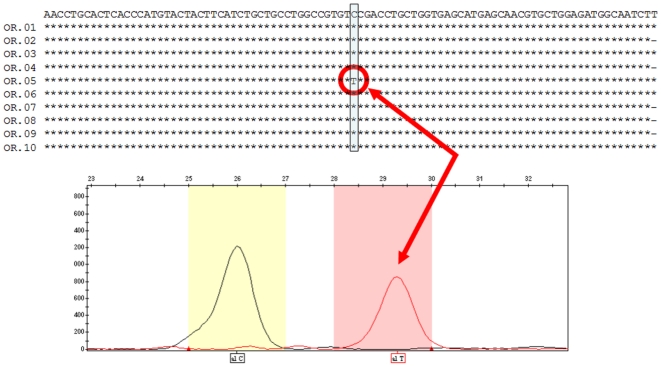
Comparison between allele frequencies of *MC1R* determined by cloning (a) and those determined by SNaPshot™ (b). Although the SNaPshot™ reaction (bottom) has isolated both peaks, cloning and sequencing (top) has reduced the T-allele to a frequency more parsimoniously attributable to a homozygous C animal where T alleles have originated from C→T transitions resulting from *post-mortem* deamination of cytosine.

The SBE protocol was faster, cheaper and more accurate than sequencing multiple clones for SNP detection in historic specimens. Since SNaPshot™ samples nearly the whole amplified molecular population, whilst cloning selects only a few molecules, SBE is far more resilient to statistical artefacts. In one case, sampling error reduced one of the two alleles to only one in ten clones (Figure 2). This frequency is more parsimoniously explained by a C→T transition lesion than by heterozygosity since this distribution is extremely unlikely if the true frequencies of the alleles are 0.5 (*p* = 0.0042 under a two-tailed t-test). Nevertheless, cloning and sequencing helped us to verify questionable or unclear SNaPshot™ results by permitting the identification and differentiation of cryptic contaminations, PCR artefacts and SNaPshot™ reaction failures.

### Future of historic and ancient SNP studies

The accuracy of our final derived SBE genotypes suggests that the potential for SNP analyses using historic and ancient DNA is great. SNP data could be used to address a wide variety of questions such as the spread of species, the prevalence of heritable disease and the domestication of plants and animals. Nevertheless, our data also reveal the pitfalls of SNP analyses. Genotyping errors, especially allelic dropout and C→T transitions, are a serious concern for any future analysis. Future studies will require extensive replication of experiments since error rates for genotypes based on single reactions are significant even in well-preserved, recent specimens.

### Conclusions

This study demonstrates that the SNaPshot™ protocol is robust for investigating phenotypic traits in historic samples. Nevertheless, SNaPshot™ results must be carefully replicated since genotypes are liable to error due to allelic dropout and C→T transitions. SBE is also a more sensitive technique than cloning and sequencing for identifying alleles. Cloning and sequencing, however, can still be useful for exploring unusual or messy SNaPshot™ results. Since SBE can be performed quickly and inexpensively by any laboratory, this technique opens past genomes to more in-depth study than has currently been achieved. This will permit us to address more detailed questions such as the prevalence of inheritable disease in the past, and phenotypic and genotypic changes resulting from processes like domestication and selective breeding.
